# Cheap Artificial Teeth, and Cheap Dental Operations in England

**Published:** 1851-04

**Authors:** 


### ARTICLE II.

### *Cheap Artificial Teeth, and Cheap Dental Operations in England.*

Whether or not we are disposed to agree with the sweeping dictum of Napoleon, that England is a nation of shopkeepers, we cannot but confess that there is no other country in the world, in which so much respect is paid to wealth, independent of rank, character or worth; or in which money so completely monopolizes every other consideration. The question generally is, not, is Mr. So-and-so a man of intellect, respectability or character? not, is he a worthy man? It usually is, does he give *noble spreads*? what is he worth?

It arises, in a great measure, from that over-respect for wealth—the *aura sacra fames*, and its concomitants—that no man sets himself down in a respectable neighborhood, with a decent establishment of servants, and displaying some kind of equipage, without exciting the curiosity and fixing the attention of his neighbors—who,, however, at once arrive at the conclusion, from the dashing and gaudy livery of the footmen, the thorough-bred bays, and the unexceptionable color of his chariot or his Brougham, that he is a visitable person; probably a brass plate on the door gives the pleasing intelligence that he rejoices in the name of either a Dobbs, Snooks or Saunders, or some other equally money-speaking patronymic, leaving the world in a pleasing uncertainty whether the numerous calls at his door are made by *mere* visitors, (in which case he must have a most extensive list of acquaintances,) or by others, who come for some more useful purpose than to kill half an hour in dissecting the characters of their neighbors, in settling how many peonies the iron duke carries in the left tail of his taglioni or paletot, or whether Miss Mullens, who lately ran away with her father's butler, was fifteen, or had arrived at the more mature age of forty, and had dyed her hair and wore false teeth.

Well, calls are made and returned—cards dropped and re-dropped—according to the usual etiquette made and provided for in these cases; each party exclaiming to their friends, oh! they are such very nice people, and so very agreeable. All goes on well and smoothly, till Dobbs, desirous of pushing his *trade*, and extending his professional connection, throws out a broad hint that he is a dentist.

The sudden explosion of a thirty-two pounder could not produce more startling effects—the news in the neighborhood travels rail road speed—one by one of poor Dobbs' pseudo-acquaintances gradually fall off—the ardent recognitions are exchanged for the cool good morning and the shoulder; before the unfortunate Dobbs has operated on a dozen grinders, he has had the cold shoulder or the cut direct from half the visiting, tea-and-turn-out portion of the neighborhood.

The junior barrister, (of twenty-five years standing, and a gentleman by act of parliament,) over the way, who lives on his wife's annuity, is obliged to allowance his family, and contracts with his tailor to be supplied with three coats, waistcoats and *continuations*, at the rate of sixteen pounds per annum—the old ones to be returned (minus the nap)—asserts his dignity.

The neighbors of equal pretensions follow his example—coolly observing, that after all, Dobbs is only a *surgeon*-dentist, and consequently not visitable. In this case, the inhabitants of —— street and its vicinity, (for it is usual to say in post octavo circulars,) feel that they have been imposed upon—that after all, Mr. Dobbs is not either a pleasant or a rich man, and that his gaudily dressed footman, and yellow chariot, and sumptuous dinner parties, are only so many fashionable traps to catch and pigeon patients.

So completely is this the case, that it was once observed by a fashionable dental practitioner—one who, by aping the most outré fastidiousness and dandyism; had managed for a time to make himself a kind of dental lion, and obtained more patients from the curiosity of the public than his own talents—that he never met a dentist, even of the first order, in the higher classes of society, or among the nobility *he visited.* It is possible, was the reply, that you are not exactly in your legitimate sphere in those places, and that the countenances of your noble hosts would be somewhat clouded by any allusion to *your trade*—nay, that you are rather endured on sufferance, than as an equal.

That the professors of the dental art are under a sort of odium, there is no doubt, or why is it that men, the highest of the class, who, by education, manners, character and attainments, are fitted for any society, all are, generally speaking, placed in the same category with the others, and considered only as dentists.

A clergyman, a lawyer and a physician have, by right of their professions, the entrée to good society, whilst the *profession* of the dentist forms rather a reason for his exclusion.

From what does this arise? Simply from this, that the dentist, (of course I speak of those educated to the profession, and dentists properly so called,) have not been true to themselves—have not unanimously asserted their dignity, but, by trusting so much to their individual respectability and talents—to the idea that their high qualifications will exempt them from a general rule, have become mixed up in the opinion of the public, with the host of the cheap advertising fraternity, who at the same time fleece their patients and disgrace the profession.

They do not appear to have read, or if they have, pay little attention to, the fable of the old man's sons and the bundle of sticks, and some others, the morals of which would point out the dangerous ground on which they stand.

It is a well known fact, that the professors of the dental art in England, form the most incongruous amalgamation of anomalies it is possible to imagine—here a ci-devant Jew clothesman, there a blacksmith in one street, and a retailer of bird's-eye and ladies' twist in another; a police dentist in one locality rivals a butcher dentist in another; even the church has furnished its contributions to the art; but "the line would stretch out to the crack of doom." Now all this would be very well, were these gentlemen to prepare themselves for their professional duties, by a proper scientific and artistic training; but, unfortunately, they seem to think that the dental art is to be acquired by mere volition, or, at the utmost, that it may be learned in a few months, or even as many days, from gentlemen who advertise to transmogrify *any* body into a full-blown dentist, in an infinitely small space of time, and compress the knowledge of five years into so many days. Some do not trouble themselves even to go through this process, but having either formed an acquaintance with some superannuated dental mechanic, and fleeced him of a little knowledge, or having been a door-porter to some dental practitioner—suddenly become accomplished dentists—their accomplishments consisting in being able to abstract a tooth some how, and plaster in a little cement *any*

*how.* Having thus fully *qualified* themselves, they commence the advertising system, and the papers, day after day, teem with their extraordinary powers and *infra*-ordinary charges—is foisted upon the unfortunate and very gullible public. The Jew-dentists offer to plaster up the carious grinders of their patrons, and make them better-ash-than-new, for two shillings each. The Christians out-doing even their Israelite brethren in rhodomontade and hyperbole and cheatery—fleecing the public till the name of dentist becomes identified with dishonesty and infamy.

It has been calculated that some of these advertisers insert four thousand notices in the course of twelve months, which, supposing each advertisement to cost five shillings, amounts to the enormous sum of £1000, and on this they anticipate, and frequently obtain, a profit of £1000 more, or, in other words, shent-per-shent on their outlay. The system must be kept up, there must be no hanging-fire, otherwise the capital originally invested will be lost. As a matter of course, these gentlemen never expect to, or are desirous of seeing their dupes a second time, taking especial care to fleece them effectually at the first. The patients have no wish for a repetition of the visit—while those patients who are not desirous of defending an action at law, on account of the absurd obloquy that is usually attached to these cases, pay the demand, however exorbitant, and think themselves extremely fortunate to have escaped from their infamous clutches.

It occasionally, however, occurs that these gentry catch a tartar, and have to make their appearance before the police magistrate. This process is the finale, and a change of name and abode becomes requisite. From Blackfriars Road, Dobbs, Snooks or a Saunders is soon transmogrified into Monsieur This, or Herr Van That, and commence again their extortions, as wholesale pseudo dental mongers.

In my next paper I shall endeavor to picture a few of the striking scenes, tricks and impositions of these worthy disciples of Scyron. R.

